# Extremely Randomized Machine Learning Methods for Compound Activity Prediction

**DOI:** 10.3390/molecules201119679

**Published:** 2015-11-09

**Authors:** Wojciech M. Czarnecki, Sabina Podlewska, Andrzej J. Bojarski

**Affiliations:** 1 Faculty of Mathematics and Computer Science, Jagiellonian University, Lojasiewicza 6, 30-348 Krakow, Poland; wojciech.czarnecki@uj.edu.pl; 2 Institute of Pharmacology, Polish Academy of Sciences, Smetna 12, 31-343 Krakow, Poland; smusz@if-pan.krakow.pl; 3 Faculty of Chemistry, Jagiellonian University, Ingardena 3, 30-060 Krakow, Poland

**Keywords:** virtual screening, compounds classification, extreme entropy machine, extremely randomized trees

## Abstract

Speed, a relatively low requirement for computational resources and high effectiveness of the evaluation of the bioactivity of compounds have caused a rapid growth of interest in the application of machine learning methods to virtual screening tasks. However, due to the growth of the amount of data also in cheminformatics and related fields, the aim of research has shifted not only towards the development of algorithms of high predictive power but also towards the simplification of previously existing methods to obtain results more quickly. In the study, we tested two approaches belonging to the group of so-called ‘extremely randomized methods’—Extreme Entropy Machine and Extremely Randomized Trees—for their ability to properly identify compounds that have activity towards particular protein targets. These methods were compared with their ‘non-extreme’ competitors, *i.e.*, Support Vector Machine and Random Forest. The extreme approaches were not only found out to improve the efficiency of the classification of bioactive compounds, but they were also proved to be less computationally complex, requiring fewer steps to perform an optimization procedure.

## 1. Introduction

Machine learning methods have recently gained extreme popularity for virtual screening tasks, providing much assistance in identifying of potentially active compounds in large chemical compound libraries. However, the increasing size of datasets, has led to higher computational expenses, and in some cases, the time needed to construct a predictive model makes a study unprofitable or even impossible because of memory limitations. To address the problem of computational expenses for large datasets in machine-learning based virtual screening, an extremely randomized learning approach was applied.

The main idea behind this family of methods is to reduce the computational and memory complexity of the statistical analysis by performing randomization instead of certain parts of an optimization procedure. For example, a nonlinear, random projection [1] could be performed instead of computing a full kernel matrix, which is required by Support Vector Machine. Another example is the random selection of the feature threshold [2].

In this study, we applied the extremely randomized learning to the problem of chemical compounds classification in order to improve the prediction accuracy and reduce the computational complexity of calculations. Two such approaches were tested: Extreme Entropy Machine (EEM) [3] and Extremely Randomized Trees (ET) [2] which were compared with the corresponding standard method—Support Vector Machine (SVM) [4] and Random Forest (RF) [5], respectively. Given the effectiveness and speed of the tested methods on one hand and huge amount of data processed in virtual screening procedures on the other, such ‘extreme’ algorithms can gain wide application in the search for new bioactive compounds.

## 2. Experimental Section

### 2.1. Datasets

The classification studies were aimed at the actives/true inactives and actives/decoys, generated according to the Directory of Useful Decoys (DUDs) procedure [6], discrimination: two sets with a different number of compounds and sets containing compounds belonging to both of these ‘inactivity’ groups—*i.e.*, mixed true inactives and DUDs; the sets were formed by merging the set of true inactives and the smaller set of DUDs (details on the compositions of particular datasets are provided in Table 1).

The ChEMBL database [7] was a source of active and inactive compounds with experimentally verified activity towards selected protein targets. The molecules for which the activity was quantified in $K_{i}$ or IC${}_{50}$ parameter were taken into account and they were considered active when the $K_{i}$ was lower than 100 nM (or IC${}_{50}$ below 200 nM) and inactive, when the $K_{i}$ was above 1000 nM (for IC${}_{50}$, the threshold was set at 2000 nM). The following targets were considered in this study: serotonin receptors 5-HT${}_{\text{2A}}$ [8], 5-HT${}_{\text{2C}}$ [9], 5-HT${}_{\text{6}}$ [10], 5-HT${}_{\text{7}}$ [11], histamine receptor H${}_{\text{1}}$ [12], muscarinic receptor M${}_{\text{1}}$ [13] and HIV related protein—HIV integrase (HIV${}_{i}$) [14].

**Table 1:** The number of compounds present in a particular dataset.

Target/Dataset	True Actives	True Inactives	DUD 1	DUD 2
5-HT${}_{2A}$	1835	851	1697	3388
5-HT${}_{2C}$	1210	926	1072	2136
5-HT${}_{6}$	1490	341	1443	2883
5-HT${}_{7}$	704	339	633	1264
M${}_{1}$	759	938	317	631
H${}_{1}$	635	545	556	1107
HIV${}_{i}$	101	914	83	163

The sets of decoys were prepared from ZINC database [15] according to the procedure described by Huang *et al.* [6]. It was preceded by the calculation for all ZINC compounds and all previously prepared sets of actives the following descriptors: logP, molecular weight (MW), number of hydrogen bond acceptors (HBA), number of hydrogen bond donors (HBD), and number of rotatable bonds (rotB) using ChemAxon tools [16]. For each considered target, the ZINC database was limited to the structures with the same number of HBA, HBD and rotB and with logP and MW values differing by no more than 10% in comparison to the active molecules. Further ZINC database narrowing was obtained by the calculation of Tanimoto coefficients towards known ligands and rejection of those structures, for which its values were higher than 0.7 (provision of physicochemical similarity and structural dissimilarity). For each set of active compounds, molecules with the lowest Tanimoto coefficient values were selected in such a number that the actives:decoys ratio was approximately 1:1 (DUD 1) and 1:2 (DUD 2).

The compounds were represented by the fingerprints generated with the PaDEL-Descriptor [17] software package: E-state Fingerprint (EstateFP, 79 bits) [18], Extended Fingerprint (ExtFP, 1024 bits) [19], Klekota and Roth Fingerprint (KlekFP, 4860 bits) [20], MACCS Fingerprints (MACCSFP, 166 bits) [21], Pubchem Fingerprint (PubchemFP, 881 bits), and Substructure Fingerprint (SubFP, 308 bits). EEM with Tanimoto projection and ET, as well as their ‘non-extreme’ competitors—SVM with radial basis kernel and RF, respectively—were applied as a classification tools with the use of the scikit-learn machine learning package. The details on the settings of each method and the ranges of parameters tested during the optimization procedure are provided in Table 2.

Balanced accuracy (BAC) was applied as the measurement of classification efficiency:

$$\mathrm{BAC}\left(\mathrm{TP},\mathrm{FP},\mathrm{TN},\mathrm{FN}\right)=\frac{1}{2}\left(\frac{\mathrm{TP}}{\mathrm{TP}+\mathrm{FN}}+\frac{\mathrm{TN}}{\mathrm{TN}+\mathrm{FP}}\right)$$

This particular statistic was selected because of the class imbalance in the datasets considered. Each of the methods tested uses an internal mechanism to maximize this statistic by weighting samples of the smaller class (SVM, RF, ET) or by being designed to address an imbalance (EEM).

**Table 2:** The range of parameters tuned during the optimization of the algorithms used. *C* hyperparameter denotes the strength of fitting to the data, *γ* is the width of the RBF kernel used in Support Vector Machine (SVM), *h* is the number of random projections (limited also by the number of training samples; in case *h* exceeded the number of examples in the training set, it was reduced to the dataset size) and ‘no of trees’, referring to the number of trees, is the size of each forest.

Method	Optimized Parameters With Range	
EEM	h∈{1000,1500,2000,2500,3000}	$C\in \{1000,10^{4},10^{5},10^{6},10^{7}\}$
SVM	γ∈{0.1,0.01,0.001,0.0001}	C∈{0.1,1,10,100,1000}
ET	no of trees ∈{10,50,100,200,500}	
RF	no of trees ∈{10,50,100,200,500}	

### 2.2. Methods

SVM is a very popular, maximum margin linear model used for binary classification. To work with non-linear decisions, a particular kernel (K) must be selected, a function that denotes the scalar product. During the optimization procedure, a training algorithm analyzes a Gram matrix (a matrix of the form $G_{ij}=K\left(x_{i},x_{j}\right)$, where $x_{i}$ is *i*th training sample), which leads to the quadratic memory requirements in terms of training set size. For a cheminformatics application, in which the number of chemical compounds can be huge [22], this becomes an impediment. At the end of the procedure, SVM reduces the number of remembered training samples via the selection of the support vectors, but during the optimization procedure, it analyzes all of them, leading to cubic computational complexity (the exact complexities of each algorithm are given in Table 3). Although it can be extremely effective in the identification of potentially active compounds, the SVM performance strongly depends on the settings under which it is run, the *C* and *γ* parameters values in particular. *C* is responsible for controlling the tradeoff between the correct classification and a large margin, whereas *γ* defines how fast RBF similarity vanishes with growing Euclidean distance between vectors.

**Table 3:** Comparison of the computational complexity of all models. *N* is the number of training samples, *d* the number of features, *h* a predefined constant (much smaller than *N*), *K* the number of trees in a forest and *k* a predefined constant (much smaller than *d*).

Method	Training Complexity	Classifying Complexity
EEM	$O(Nh^{2})$	O(hd)
SVM	$O(N^{3})$	O(Nd)
ET [2]	O(KkNlogN)	O(Kk)
RF [2]	O(KdNlogN)	O(Kd)

In EEM, this restriction of analyzing all the samples is removed by the introduction of random projections in place of the kernel. A possible method used in this paper for defining such random projections is the random selection of a subset of the training samples and the subsequent computation of only part of the original Gram matrix (*i.e.*, only the columns corresponding to the selected compounds). Furthermore, entropy based optimization is performed in the new, random projected space, which can be solved extremely quickly ($O(Nh^{2})$, where *h* is the number of selected compounds and *N* is the size of the training set). Contrary to SVM, EEM has a closed form solution of the optimization problem, which makes the return of an exact solution much more probable (SVM has a convex optimization function, meaning that optimization converges to a global optimum; however, due to numerical errors and stability, it often stops before the true solution is achieved).

In summary, two main differences exist between SVM and EEM. First, SVM fully optimizes which samples become support vectors, which is expensive both computationally and in terms of memory. EEM uses randomization to limit the set that might be used as a support vector competitor (*i.e.*, the base of the projected space) and performs the optimization later on. Consequently EEM is a much more efficient approach. A second difference arises from a different formulation of the final optimization procedure, which, despite similarities [3], is much simpler and can be solved orders of magnitude more quickly.

RF is currently one of the most successful out of the box methods for building classifiers [23]. It grows a set of decision trees, modified in two significant ways. First, in each internal node, only a random subset of all the features is considered, which helps the model not to overfit. Later, during the optimization procedure, an optimal threshold to split the training set is selected, creating a decision rule. Second, each tree works with a slightly different training set, which is achieved by the introduction of bagging, in which training sets are constructed by sampling with replacement from the original training set. These two small modifications lead to a significant increase in the generalization capability. The final prediction for a given sample is the averaged prediction from all individual trees.

However, it appears that the model can be strengthened even further by randomization of the threshold selection for each decision rule. Instead of performing internal optimization, thresholds are simply selected at random, and the best one is chosen. This slight modification leads to the construction of the ET model and even better generalization abilities with the simultaneous reduction of the computational complexity of the model.

For both SVM and EEM, as well as for RF and ET, the ‘extreme’ counterpart changes an optimization element into a randomized process. Although it might be counterintuitive that random action could be better than a well-optimized approach, it is a common phenomenon in machine learning [1,24,25].

**Figure 1:**
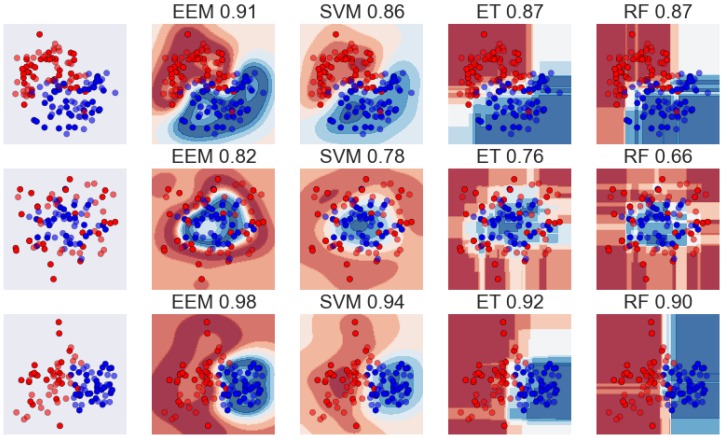
Comparison of the classification of simple 2D datasets with all models used. The number above a particular picture denotes the generalization accuracy.

A sample analysis of the decision boundaries arrived at by each of the methods tested and their generalization abilities are shown in Figure 1. This figure shows three simple, two-dimensional datasets split randomly into training and test sets (in a 1:1 proportion) that are modeled using each of the methods described (SVM and EEM use the exact same hyperparameters, as do RF and ET). In each example, the ‘extreme’ method achieves a higher generalization score. Furthermore, EEM builds much more general decision boundaries than SVM (which allows better density estimation), thus confirming earlier claims about the use of randomization to address overfitting. ET, in contrast, builds ‘smoother’ decision boundaries than RF, again because of high randomization, and consequently has better generalization capabilities.

## 3. Results and Discussion

The following aspects were the main focus for the analysis of the results: the effect of the application of the extreme approach on the classification effectiveness and the computational complexity of the algorithms used in the study together with the difficulty of their optimization procedure. The results were compared for the optimal conditions for the particular set of experiments (protein/representation).

The analysis of both training and classifying complexity (Table 3) indicates that the extreme approaches are less complex than the corresponding standard methods for both of these comparisons. The training complexity of EEM is much lower for both of the analyzed parameters (training and classification) and is equal to $O(Nh^{2})$ and O(hd), respectively, whereas for SVM, it is $O(N^{3})$ and O(Nd), where *N* is the number of training samples and *h* is a predefined constant that is much smaller than *N*. When ET is compared with RF, it has O(KkNlogN) training and O(Kk) classifying complexity for ET, and the competitors for RT are equal to O(KdNlogN) and O(Kd) for training and classification, respectively, where *K* is the number of trees in a forest and *k* is analogous to *h* and is a predefined constant much smaller than *d*.

The detailed results for the selected sets of experiments (discrimination between actives and two groups of inactives—true inactives and DUDs) are presented in Table 4 (the results for the other datasets are placed in the Supporting Information). This is a global analysis of the results; *i.e.*, all the methods are presented simultaneously. The highest BAC values obtained for a particular target/fingerprint pair are marked with an asterisk sign, whereas the winner of a particular pair (EEM-SVM and ET-RF) is indicated in bold.

In general, the classification accuracy was very high, with BAC values exceeding 0.9 in the majority of cases. Depending on the fingerprint, the most effective method varied: EEM provided the highest BAC values of all the tested methods—for MACCSFP for all targets considered, for SubFP for all but one protein, for 5 of 7 targets for PubchemFP and for 4 of 7 targets when KlekFP was used to representat the compounds. For the other fingerprints the results varied—for EstateFP, EEM and ET provided the highest BAC values for 3 proteins, whereas SVM and RF won only once. In contrast, when compounds were represented by ExtFP, SVM provided the highest number of best BAC values (4), but the other three experiments were won by EEM. When the ‘extreme’ and standard approaches were compared, in general, the former methods gave higher BAC values than their ‘non-extreme’ competitors (as indicated values in bold). For some fingerprints, when the classification effectiveness was very high, some draws occurred (their higher number was observed for PubchemFP, in which differences in BAC values were obtained only for M_1_ and HIV${}_{i}$ for the ‘extreme’ and ‘non-extreme’ approaches). However, for all the remaining fingerprints, a clear advantage of EEM and ET over SVM and RF, respectively, was observed. Because the BAC values were already very high in most cases (greater than 0.95), the improvement gained from the ‘extreme’ approach was not much, usually no more than 1 percentage point. However, other features, such as the computational complexity and the simple optimization procedure, make EEM and ET preferable to the standard methods.

The results were also analyzed in a slightly different, non-standard manner. Table 5 shows the results for all datasets and methods, in a way, that the method ‘chooses’ the best representation of the compounds and all fingerprints are considered simultaneously. In this case, the classification is much more effective, with BAC values approaching or equal to 1 for experiments discriminating actives from DUDs and over 0.9 or close to this threshold for the majority of actives/true inactives experiments. For both the DUDs datasets, the ‘extreme’ approaches also provided the highest BAC scores in the majority of cases—EEM in 4 of 7 cases and ET in 5 of 7 cases for the first DUDs dataset and both of these methods for all but one target in the extended DUDs dataset. When active compounds were identified among true inactives, EEM was the most effective approach for 5 of 7 proteins, and when the set of inactives was formed both by true inactives and DUDs, this method was the best in 4 of 7 cases. A pairwise comparison between EEM/SVM and ET/RF revealed that EEM surpassed SVM in the majority of cases and that ET surpassed RF for most of the target/fingerprint combinations. For actives/DUDs recognition, EEM and ET surpassing SVM and RF occurred in all the cases (including draws), whereas when the set of inactives also contained some true inactive molecules, ‘extreme’ methods won in 4 of 7 (EEM) and in 3 of 7 trials (ET), plus one draw that occurred in the latter case.

**Figure 2:**
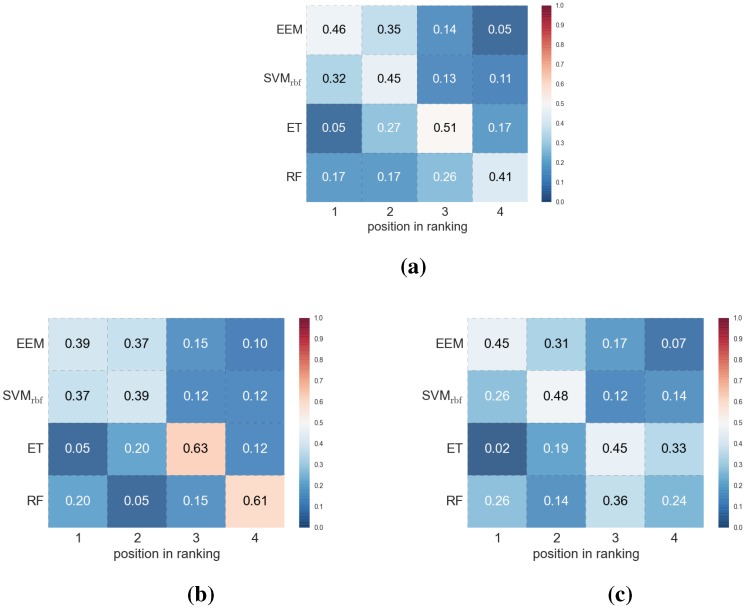

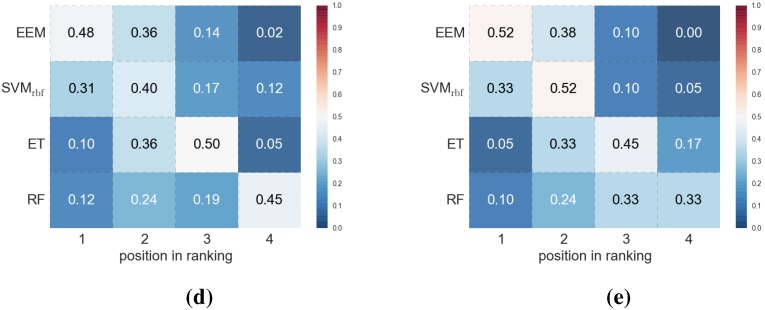
Heat maps that visualize the probability of a given method being at a particular position in ranking: (**a**) All experiments together; (**b**) actives/true inactives dataset; (**c**) actives/true inactives + DUDs dataset; (**d**) actives/DUD 1 dataset; (**e**) actives/DUD 2 dataset.

We conducted an additional analysis, an empirical estimation of the position in the ranking in which a particular machine learning method is placed (the ranking refers here to the arrangement of methods according to the decreasing BAC values). Figure 2, shows heat maps with probabilities that a particular method would assume a particular position in such ranking when all experiments were taken into account and when each particular dataset was considered separately. All the heat maps clearly indicate that EEM is most likely to provide the highest classification efficiency—in all the situations considered, the probability that the best results would be obtained by this method was the highest, with the second position in the ranking being the runner-up in all cases.

**Figure 3:**
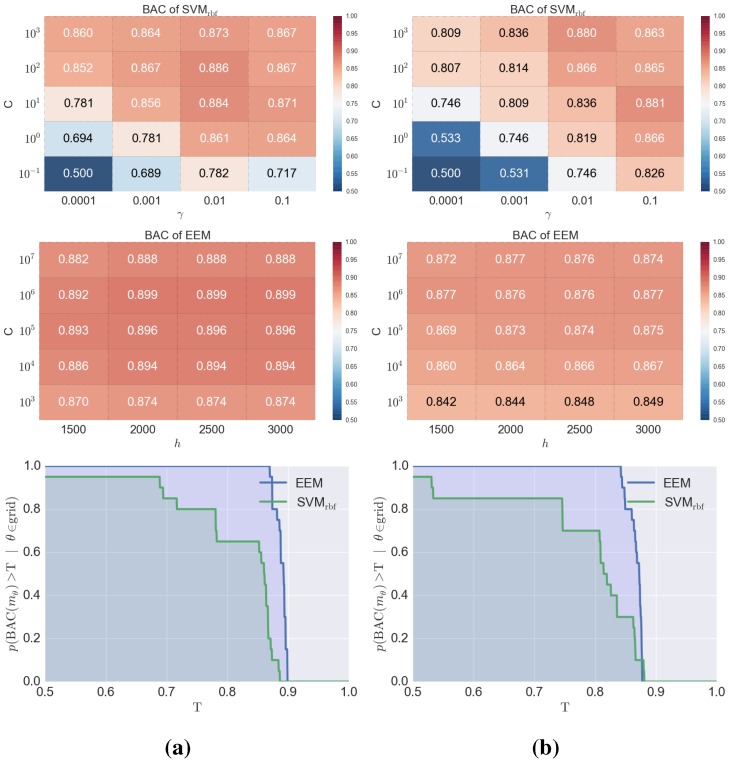
Visualization of the optimization procedure for Support Vector Machine (SVM) and Extreme Entropy Machine (EEM) for the selected datasets: (**a**) M${}_{1}$ with KlekFP; (**b**) 5-HT${}_{2A}$ with SubFP. In the top rows—BAC scores obtained for a particular set of hyperparameters values during a grid search. At the bottom—a plot of the probabilities of obtaining at least a given BAC score (T) from a given model assuming a random selection of hyperparameters from the grid of hyperparameters used.

**Figure 4:**
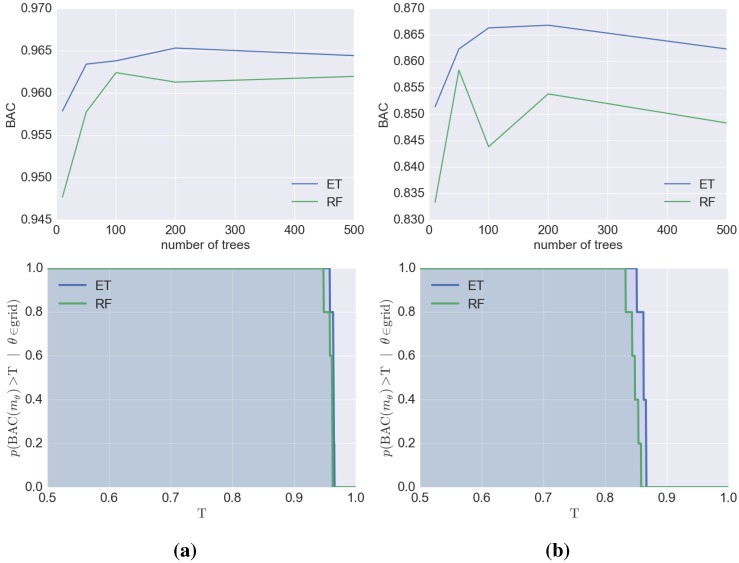
Visualization of the optimization procedure of ET and RF for selected datasets: (**a**) 5-HT${}_{6}$ with ExtFP; (**b**) HIV${}_{i}$ with PubchemFP. In the top row—BAC scores obtained for a particular number of trees. In the bottom row—a plot of the probabilities of obtaining at least a given BAC score (T) from a given model assuming a random selection of the number of trees from the tested range.

Finally, for the selected target/fingerprint combinations, the methods were compared in terms of the difficulty of finding optimal parameters—EEM and SVM are shown in Figure 3 and ET and RF in Figure 4. Both figures show examples of target/fingerprint pairs; all the remaining data are in the Supporting Information. Both types of analyses clearly indicate that the optimization of EEM is much easier than that of SVM. Not only are the BAC scores obtained for particular sets of parameters tested higher for EEM than SVM, but it is also noteworthy that, in general, EEM is a much more stable method than SVM in terms of the prediction efficiency and can be considered as safer for unexperienced users—the variability of BAC values are significantly lower for EEM, whereas for SVM, improper conduct of the optimization procedure could lead to BAC values as low as 0.5. A similar conclusion can be drawn from the ET/RF comparison in which a number of trees was optimized during the training procedure. The top portion of the Figure 4 indicates that the BAC values depend on the number of trees—in both cases analyzed, the BAC values for ET were significantly higher for both target/fingerprint examples. Moreover, ET is also much more stable (similar to EEM), when the number of trees is changed—the BAC values changed by up to 15% for ET, but for RF, the BAC values changed by approximately 25% when the number of trees was varied. A similar situation occurred, when the probability of obtaining at least a given BAC score for each model was analyzed, although, the difference between ET and RF is not as evident in this case, but the probabilities are slightly higher (1-2 percentage points) for ET.

**Table 4:** BAC results for actives/true inactives and DUDs datasets. Asterisk indicates the highest BAC values obtained for a particular target/fingerprint pair.

EstateFP	EEM	SVM${}_{\mathrm{rbf}}$	ET	RF	SubFP	EEM	SVM${}_{\mathrm{rbf}}$	ET	RF
5-HT${}_{2A}$	${}^{*}$0.936	0.928	0.932	**0.935**	5-HT${}_{2A}$	${}^{*}$0.968	0.964	**0.967**	0.964
5-HT${}_{2C}$	0.913	**0.920**	0.923	${}^{*}$0.927	5-HT${}_{2C}$	${}^{*}$0.951	0.945	0.935	**0.940**
5-HT${}_{6}$	**0.964**	0.961	${}^{*}$0.967	0.965	5-HT${}_{6}$	${}^{*}$0.984	0.980	**0.982**	0.981
5-HT${}_{7}$	${}^{*}$0.925	0.920	${}^{*}$0.925	0.922	5-HT${}_{7}$	${}^{*}$0.976	0.974	${}^{*}$0.976	${}^{*}$0.976
M${}_{1}$	${}^{*}$0.925	0.917	**0.916**	0.912	M${}_{1}$	${}^{*}$0.967	0.965	**0.965**	0.960
H${}_{1}$	**0.922**	0.918	${}^{*}$0.927	0.925	H${}_{1}$	${}^{*}$0.970	0.968	**0.967**	0.965
HIV${}_{i}$	0.968	${}^{*}$0.983	**0.971**	**0.971**	HIV${}_{i}$	**0.980**	**0.980**	${}^{*}$0.985	${}^{*}$0.985
PubchemFP	EEM	SVM${}_{\mathrm{rbf}}$	ET	RF	ExtFP	EEM	SVM${}_{\mathrm{rbf}}$	ET	RF
5-HT${}_{2A}$	${}^{*}$0.999	${}^{*}$0.999	${}^{*}$0.999	${}^{*}$0.999	5-HT${}_{2A}$	${}^{*}$0.986	0.982	**0.979**	0.975
5-HT${}_{2C}$	**0.999**	**0.999**	${}^{*}$1.000	${}^{*}$1.000	5-HT${}_{2C}$	${}^{*}$0.982	0.980	**0.981**	0.979
5-HT${}_{6}$	${}^{*}$0.999	${}^{*}$0.999	${}^{*}$0.999	${}^{*}$0.999	5-HT${}_{6}$	0.991	${}^{*}$0.992	**0.989**	0.988
5-HT${}_{7}$	${}^{*}$0.999	${}^{*}$0.999	${}^{*}$0.999	${}^{*}$0.999	5-HT${}_{7}$	0.981	${}^{*}$0.982	**0.978**	0.977
M${}_{1}$	**0.996**	0.994	${}^{*}$0.998	0.995	M${}_{1}$	${}^{*}$0.976	0.973	**0.971**	0.965
H${}_{1}$	${}^{*}$1.000	${}^{*}$1.000	**0.999**	**0.999**	H${}_{1}$	0.972	${}^{*}$0.977	**0.967**	0.962
HIV${}_{i}$	${}^{*}$1.000	0.994	**0.995**	0.990	HIV${}_{i}$	0.984	${}^{*}$0.990	**0.980**	**0.980**
KlekFP	EEM	SVM${}_{\mathrm{rbf}}$	ET	RF	MACCSFP	EEM	SVM${}_{\mathrm{rbf}}$	ET	RF
5-HT${}_{2A}$	${}^{*}$0.992	0.988	**0.986**	0.983	5-HT${}_{2A}$	${}^{*}$0.984	0.981	**0.978**	0.976
5-HT${}_{2C}$	${}^{*}$0.991	0.986	**0.980**	0.975	5-HT${}_{2C}$	${}^{*}$0.982	0.977	**0.975**	0.972
5-HT${}_{6}$	**0.999**	**0.999**	${}^{*}$1.000	${}^{*}$1.000	5-HT${}_{6}$	${}^{*}$0.988	${}^{*}$0.988	**0.981**	0.979
5-HT${}_{7}$	0.987	${}^{*}$0.989	**0.981**	0.980	5-HT${}_{7}$	${}^{*}$0.982	0.975	**0.979**	0.975
M${}_{1}$	${}^{*}$0.977	0.974	**0.964**	0.956	M${}_{1}$	${}^{*}$0.975	${}^{*}$0.975	**0.965**	0.962
H${}_{1}$	${}^{*}$0.987	0.981	**0.986**	0.984	H${}_{1}$	${}^{*}$0.975	${}^{*}$0.975	**0.974**	**0.974**
HIV${}_{i}$	**0.984**	0.980	0.984	${}^{*}$0.989	HIV${}_{i}$	${}^{*}$0.989	0.984	**0.984**	0.978

**Table 5:** Comparison of the BAC scores obtained for each experiment, in which the method chooses the best fingerprint. Asterisk indicates the highest BAC values obtained for a particular target.

True Inactives	EEM	SVM${}_{\mathrm{rbf}}$	ET	RF	trueInact/DUDs	EEM	SVM${}_{\mathrm{rbf}}$	ET	RF
5-HT${}_{2A}$	${}^{*}$ 0.882	0.875	**0.862**	0.852	5-HT${}_{2A}$	0.918	${}^{*}$0.919	0.917	**0.918**
5-HT${}_{2C}$	0.875	${}^{*}$0.885	**0.883**	0.881	5-HT${}_{2C}$	${}^{*}$0.904	0.899	**0.901**	0.895
5-HT${}_{6}$	${}^{*}$0.901	0.895	**0.888**	0.885	5-HT${}_{6}$	0.965	${}^{*}$0.967	**0.965**	0.962
5-HT${}_{7}$	${}^{*}$0.876	0.868	**0.847**	0.825	5-HT${}_{7}$	${}^{*}$0.924	0.921	**0.907**	**0.907**
M${}_{1}$	${}^{*}$0.888	0.882	0.885	**0.887**	M${}_{1}$	${}^{*}$ **0.899**	0.890	0.890	**0.893**
H${}_{1}$	${}^{*}$0.919	0.913	0.908	**0.911**	H${}_{1}$	${}^{*}$0.928	0.923	0.926	**0.927**
HIV${}_{i}$	0.911	${}^{*}$0.920	**0.867**	0.859	HIV${}_{i}$	0.899	${}^{*}$0.919	**0.867**	0.858
DUD 1	EEM	SVM${}_{\mathrm{rbf}}$	ET	RF	DUD 2	EEM	SVM${}_{\mathrm{rbf}}$	ET	RF
5-HT${}_{2A}$	${}^{*}$ **0.999**	${}^{*}$0.999	${}^{*}$0.999	${}^{*}$0.999	5-HT${}_{2A}$	${}^{*}$0.999	${}^{*}$0.999	${}^{*}$0.999	${}^{*}$0.999
5-HT${}_{2C}$	**0.999**	**0.999**	${}^{*}$1.000	${}^{*}$1.000	5-HT${}_{2C}$	${}^{*}$1.000	0.999	${}^{*}$1.000	${}^{*}$1.000
5-HT${}_{6}$	**0.999**	**0.999**	${}^{*}$1.000	${}^{*}$1.000	5-HT${}_{6}$	**0.999**	**0.999**	${}^{*}$1.000	${}^{*}$1.000
5-HT${}_{7}$	${}^{*}$0.999	${}^{*}$0.999	${}^{*}$0.999	${}^{*}$0.999	5-HT${}_{7}$	${}^{*}$0.999	${}^{*}$0.999	${}^{*}$0.999	${}^{*}$0.999
M${}_{1}$	**0.996**	0.994	${}^{*}$0.998	0.995	M${}_{1}$	${}^{*}$0.996	0.994	${}^{*}$0.996	${}^{*}$0.996
H${}_{1}$	${}^{*}$1.000	${}^{*}$1.000	**0.999**	**0.999**	H${}_{1}$	${}^{*}$1.000	${}^{*}$1.000	${}^{*}$1.000	0.999
HIV${}_{i}$	${}^{*}$1.000	0.994	**0.995**	0.990	HIV${}_{i}$	${}^{*}$0.995	${}^{*}$0.995	**0.990**	**0.990**

## 4. Conclusions

In this study, new types of algorithms were introduced for the tasks connected with the evaluation of the biological activity of chemical compounds—Extreme Entropy Machine and Extremely Randomized Trees. Both methods were compared with their ‘non-extreme’ analogues—Support Vector Machine and Random Forest, respectively. The results indicated that EEM and ET performed better than their ‘non-extreme’ competitors: SVM and RF, respectively. EEM and ET were also proved to be less computationally complex. Moreover, a careful analysis of the course of the optimization procedure for both of these algorithms showed the significant simplicity of both of the ‘extreme’ approaches tested and less variability in the predictive power of the models depending on the values of the optimized parameters. Because virtual screening procedures use a high amount of data and the libraries evaluated by this approach often contain an enormous number of structures, the computational simplifications and ease of performing the optimization procedure make the ‘extreme’ approaches tested valuable methods for tasks connected with the search for new bioactive compounds in large libraries of molecules.
